# Clinicopathological Features and Prognostic Factors of Colorectal Neuroendocrine Neoplasms

**DOI:** 10.1155/2017/4206172

**Published:** 2017-01-17

**Authors:** Mengjie Jiang, Yinuo Tan, Xiaofen Li, Jianfei Fu, Hanguang Hu, Xianyun Ye, Ying Cao, Jinghong Xu, Ying Yuan

**Affiliations:** ^1^Department of Medical Oncology, The Second Affiliated Hospital, Zhejiang University School of Medicine, Hangzhou, Zhejiang Province, China; ^2^Department of Surgical Oncology, The Second Affiliated Hospital, Zhejiang University School of Medicine, Hangzhou, Zhejiang Province, China; ^3^Cancer Institute (Key Laboratory of Cancer Prevention and Intervention, Chinese National Ministry of Education; Key Laboratory of Molecular Biology in Medical Sciences, Zhejiang Province, China), The Second Affiliated Hospital, Zhejiang University School of Medicine, Hangzhou, Zhejiang Province, China; ^4^Department of Oncology, Jinhua Central Hospital (Jinhua Hospital of Zhejiang University School of Medicine), Jinhua, Zhejiang Province, China; ^5^Department of Pathology, The Second Affiliated Hospital, Zhejiang University School of Medicine, Hangzhou, Zhejiang Province, China

## Abstract

*Background*. Limited research is available regarding colorectal NENs and the prognostic factors remain controversial.* Materials and Methods*. A total of 68 patients with colorectal NENs were studied retrospectively. Clinical characteristics and prognosis between colonic and rectal NENs were compared. The Cox regression models were used to evaluate the predictive capacity.* Results*. Of the 68 colorectal NENs patients, 43 (63.2%) had rectal NENs, and 25 (36.8%) had colonic NENs. Compared with rectal NENs, colonic NENs more frequently exhibited larger tumor size (*P* < 0.0001) and distant metastasis (*P* < 0.0001). Colonic NENs had a worse prognosis (*P* = 0.027), with 5-year overall survival rates of 66.7% versus 88.1%. NET, NEC, and MANEC were noted in 61.8%, 23.5%, and 14.7% of patients, respectively. Multivariate analyses revealed that tumor location was not an independent prognostic factor (*P* = 0.081), but tumor size (*P* = 0.037) and pathological classification (*P* = 0.012) were independent prognostic factors.* Conclusion*. Significant differences exist between colonic and rectal NENs. Multivariate analysis indicated that tumor size and pathological classification were associated with prognosis. Tumor location was not an independent factor. The worse outcome of colonic NENs observed in clinical practice might be due not only to the biological differences, but also to larger tumor size in colonic NENs caused by the delayed diagnosis.

## 1. Introduction

Neuroendocrine neoplasms (NENs) consist of a spectrum of malignancies that arise from neuroendocrine cells, which are located throughout the body. NENs are a group of fairly rare tumors with obvious heterogeneity. These tumors were formerly referred to as “carcinoid,” which means “carcinoma-like.”

Based on the current literature, the worldwide incidence of NENs seems to have increased markedly [1–4]. According to the Surveillance Epidemiology and End Results (SEER) database of the United States, the annual incidence of NENs has increased nearly fivefold from 1973 (1.09/100,000) to 2004 (5.25/100,000) [5]. More than half of all NENs are gastroenteropancreatic NENs (GEP-NENs), with an annual incidence of 3.65–4.7/100,000 in the United States [5–7]. The annual incidence of colonic NENs increased from 0.02/100,000 to 0.2/100,1000 and rectal NENs increased from 0.2/100,000 to 0.86/100,000 [8]. Widespread endoscopic screening, increased awareness of neuroendocrine histology, and improved data capture likely have contributed to this trend [4, 5, 9–11]. The most common primary site of colonic NENs is the cecum, followed by sigmoid colon and ascending colon. Rectal NENs occurred at a markedly higher frequency among Asian population than among white patients [5, 8, 12, 13].

The nomenclature and classification of NENs have always been a dispute, which are lack of uniform standard. Traditionally, NENs were classified based on the embryonic origins as foregut, midgut, and hindgut tumors. However, recent attempts have been made to change the nomenclature according to primary sites [8]. The World Health Organization (WHO) classifies NENs of the colon and rectum together as a single entity. According to the 2010 WHO classification, NENs are classified as neuroendocrine tumor (NET), neuroendocrine carcinoma (NEC), or mixed adenoneuroendocrine carcinoma (MANEC). Furthermore, NET/NEC are graded into three levels based on different definitions of proliferation using the mitotic count and/or the Ki-67 index: Grade 1 (G1), mitotic count < 2 per 10 high-power fields (HPF) and/or Ki-67 ≤ 2%; Grade 2 (G2), mitotic count 2–20 per 10 HPF and/or Ki-67 3 to 20%; Grade 3 (G3), mitotic count > 20 per 10 HPF and/or Ki-67 > 20% [8, 14].

It is generally accepted that tumor size and pathological classification are associated with prognosis [5, 12, 14, 15]. However, the impact of tumor location on the outcome of colorectal NENs remains controversial. Clinical practice demonstrated a better outcome in rectal NENs compared with colonic NENs, but whether tumor location is an independent prognostic factor remains unknown. It is not clear whether differences in prognosis between colonic NENs and rectal NENs are due to inherent distinctions between these conditions. Given the significant differences in prognosis between colonic NENs and rectal NENs, should different treatments be administered to these two groups?

Many American and European studies have reported the epidemiology, clinical manifestations, pathology, management, and survival of GEP-NENs [5, 7, 9], but there is a lack of data in Asian population, especially in China. And the research about colorectal NENs is rare because of the low incidence. Therefore, the objective of the present study was to perform an epidemiological and prognosis research of colorectal NENs in a Chinese population [16]. We collected and retrospectively analyzed the data from colorectal NEN patients registered at the Second Affiliated Hospital, Zhejiang University School of Medicine, from March 2001 to March 2014 to investigate the clinicopathological characteristics and prognostic factors of colorectal NENs.

## 2. Materials and Methods

A total of 68 colorectal NENs patients who were treated in the Second Affiliated Hospital, Zhejiang University School of Medicine, between March 2001 and March 2014 were included in this retrospective study. All of the cases were confirmed by pathology. To standardize the pathological diagnosis, the same pathologist reviewed all slides. The hospital's ethics committee approved this study with written informed consent. Data obtained from the patients included demographic (e.g., age, gender, and diagnosed time), clinical (e.g., symptoms at presentation, tumor location, treatment, and survival time), and pathological data (e.g., tumor size, depth of invasion, lymph nodes status, distant metastasis, pathological classification, and Ki-67 index).

Tumors were restaged according to the American Joint Committee on Cancer (AJCC) 7th Tumor, Lymph Node and Metastasis (TNM) staging system. The tumor location was described as colon or rectum. The rectum was defined as being 15 centimeters from the anal verge. Both the sigmorectal junction and ileocecal junction were classified as the colon. Classification and grading were based on morphological criteria and tumor proliferative activity according to the 2010 WHO classification. In treatment, patients were classified as undergoing regional surgery, endoscopic radical surgery, or best supportive care. The regional surgeries encompass anterior resection, abdominal perineal resection, and transanal endoscopic microsurgery (TEM), and endoscopic radical surgeries encompass endoscopic mucosal resection (EMR) and endoscopic submucosal dissection (ESD). Best supportive care means cancer pain control, nutritional support, and symptomatic treatment.

Specific staff members in the oncology institution were responsible for collecting data from patients and subsequently contacting with patients. Follow-up was conducted by a combination of physical examination, colonoscopy, and computed tomography at either six-monthly or yearly intervals. Overall survival was calculated from the time of the patient's final diagnosis to their death caused by colorectal NENs. Death attributed to other causes or patients lost to follow-up were defined as censored observation.

Data of all categorical variables were summarized using frequencies and percentages. Comparisons between groups were performed using Pearson's chi-square or Fisher's exact tests. Survival curves were generated using Kaplan-Meier methods, and the log-rank test was performed to evaluate the survival difference. Adjusted relative ratios (RRs) along with 95% confidence intervals (CI) were calculated using Cox proportional hazards regression models. When the two-side *P* value was less than 0.05, the difference was considered statistically significant. SPSS 16.0 statistics software (SPSS Chicago IL, USA) was used for data analysis.

## 3. Results

### 3.1. Clinicopathological Features of the 68 Patients

The current cohort represented 68 adult patients with colorectal NENs. Of these patients, 43 (63.2%) had rectal NENs, and 25 (36.8%) had colonic NENs. All the patients were Han Chinese. Additionally, 44 (64.7%) patients were male, and 24 (35.3%) were female. The male-to-female ratio was 1.8 : 1. The average age was 55.7 years old (range, 20 to 82 years old). Among all the patients, 25 (36.8%) patients presented with abdominal pain, 11 (16.2%) with hematochezia, 4 (5.8%) with an alteration in stool property, 3 (4.4%) with an alteration in bowel habit, and 1 (1.5%) with unexplained weight loss. The remaining 24 (35.3%) cases were an incidental finding without obvious symptoms. None of the patients presented with carcinoid syndrome (e.g., hot flash, watery diarrhea, or palpitation). No synchronic NEN was noted in other parts of the body. For rectal NENs, the median distance from the anal verge was 7.0 cm (range, 2 to 15 cm).

All 68 patients were diagnosed via histopathology. The median diameter on histological analysis was 10 mm (range, 2 to 200 mm). Moreover, 30 lesions (44.1%) were smaller than 10 mm in diameter, 8 lesions (11.8%) ranged from 11 to 20 mm, and 30 lesions (44.1%) were larger than 20 mm. According to the 2010 WHO classification, 42 of 68 (61.8%) cases were classified as NET, 16 (23.5%) as NEC, and 10 (14.7%) as MANEC. Some pathology reports (*n* = 19) did not present the Ki-67 index. According to the available data (*n* = 49), the Ki-67 indices of 27 (39.7%) patients were ≤2%, 6 (8.8%) ranged from 3% to 20%, and 16 (23.5%) were >20%. Mitotic rates were not reported in most pathology reports.

### 3.2. Distinctions between Colonic NENs and Rectal NENs

Significant differences were noted between colonic NENs and rectal NENs in clinical practice. Rectal NENs exhibited increased morbidity compared with colonic NENs. The latter cecum was the most common site involved followed by the ascending colon and sigmoid colon. In addition, rectal NENs were often diagnosed in patients of a relatively younger age (*P* = 0.01).

Rectal NENs were typically smaller than colonic tumors (*P* < 0.0001) and always located on the anterior or lateral rectal wall. A significant difference was noted between colonic NENs and rectal NENs regarding pathological classification (*P* = 0.001). More rectal NENs were classified as well-differentiated NET, whereas more colonic NENs were poorly differentiated NEC/MANEC. A similar trend was observed regarding tumor stage and tumor grade; namely, colonic NENs were often diagnosed at later stage (*P* < 0.0001) and higher grade. Compared with rectal NENs, colonic NENs were more likely metastatic when diagnosed. A total of 18 (26.5%) patients had metastases at the time of diagnosis. Of these patients, 13 had colonic NENs. Metastases were often noted in the liver, lymph nodes, and mesenteric peritoneum.

In summation, colonic NENs were relatively scarce compared with rectal NENs but occurred at a markedly increased frequency with larger tumor size, poorly differentiated classification, and distant metastases. The detailed distinctions between rectal NENs and colonic NENs are provided in Table 1.

### 3.3. Therapy

The majority of the patients (*n* = 52) underwent regional surgery with curative intent (*n* = 47) or for palliative purposes (*n* = 5). A total of 4 patients with metastatic disease underwent resection of their metastatic lesions, including liver metastasis, gallbladder metastasis, and adnexa metastasis. A total of 15 patients underwent endoscopic radical surgery, among which 12 patients underwent complete excision with a negative margin and 3 patients were with a positive margin; no specimens were fragmented. Only one patient pathologically diagnosed via endoscopic biopsy did not undergo surgical operation due to the presence of widespread metastases and poor physical condition. He received best supportive care exclusively. There are 3 patients who took preoperative chemotherapy and 5 patients took postoperative adjuvant chemotherapy. Palliative chemotherapy was administered to 5 patients. The chemotherapy regimens included oxaliplatin-fluorouracil (*n* = 2), platinum-etoposide (*n* = 2), and irinotecan-fluorouracil (*n* = 1). None of the patients received radiotherapy and targeted therapy.

### 3.4. Survival and Prognostic Factors

The cut-off date of follow-up was September 2014. In total, 63 of 68 patients received complete follow-up with a median duration of 4 years (range, 0.5 to 13 years). The 1-year, 3-year, and 5-year survival rates of the entire cohort were 89.7%, 85.3%, and 82.4%, respectively.

### 3.5. Univariate Analyses of Outcome

**Figure 1:**
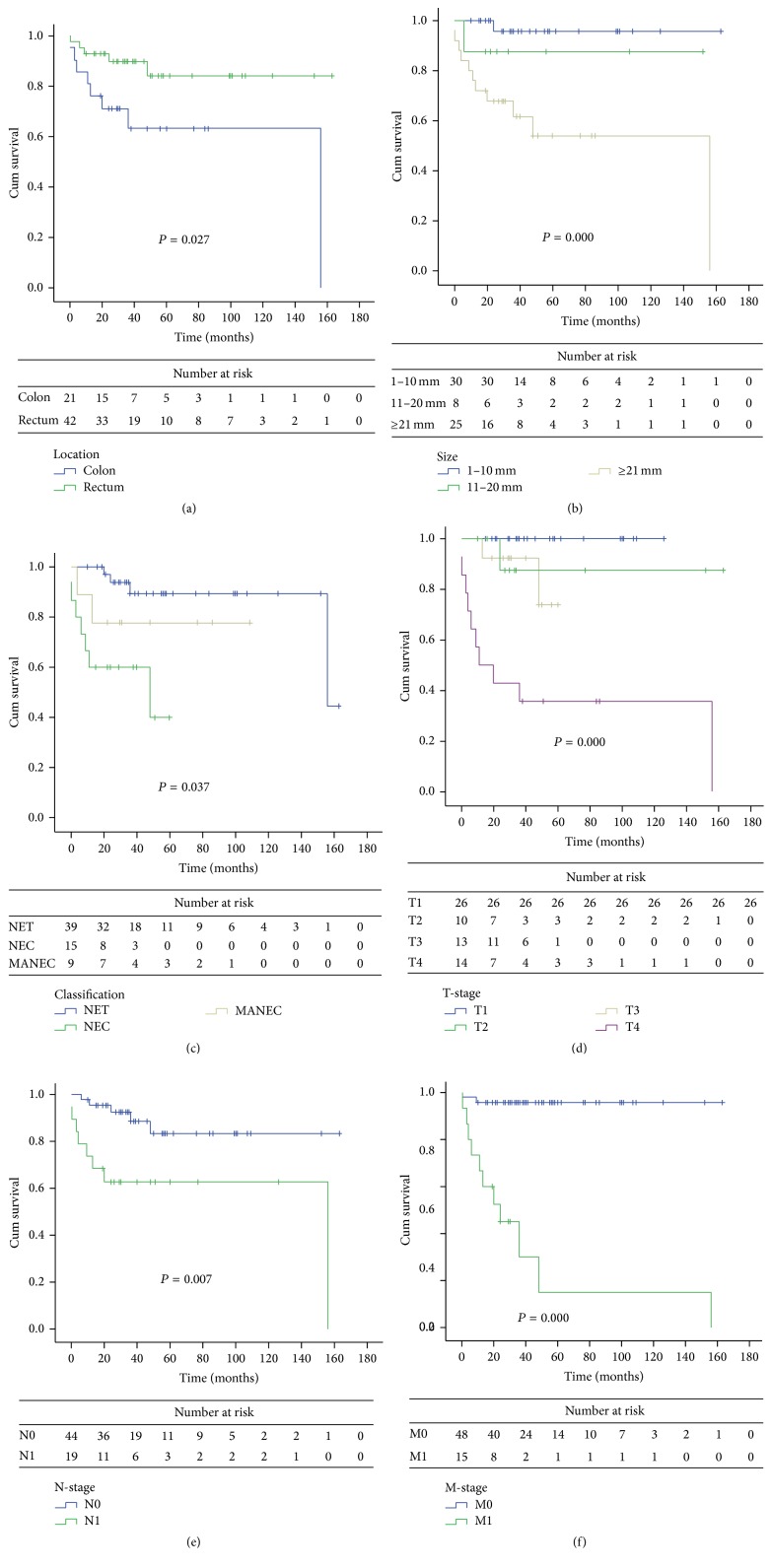
(a) Overall survival based on tumor location. (b) Overall survival based on tumor size. (c) Overall survival based on pathological classification. (d) Overall survival based on T-classification. (e) Overall survival based on N-classification. (f) Overall survival based on M-classification.

Regarding the impact of tumor location on outcome, univariate analysis indicated that the colonic NENs exhibited worse outcomes compared with rectal NENs. The 5-year overall survival rates were 66.7% and 88.1% for colonic NENs and rectal NENs subgroups, respectively, and a significant difference was noted (*P* = 0.03) (Figure 1(a)).

Additionally, tumor size, pathological classification, tumor infiltration (T-classification), lymph nodes status (N-classification), and distant metastasis (M-classification) could predict the outcome, whereas the age or the gender could not. Larger tumor size, poorly differentiated pathological classification (NEC/MANEC), T-classification (T3/T4), N-classification (N1), and M-classification (M1) were associated with dismal prognoses (*P* < 0.05). The 5-year survival rate was 33.3% in patients with distant metastases and 95.8% in patients without distant metastases (Figure 1).

### 3.6. Multivariate Analyses of Outcome

All factors associated with survival based on univariate analysis and various innate factors, such as gender and age, were included in the Cox model. In the multivariate analysis, tumor size, pathological classification, age, T-classification, and M-classification were independent prognostic factors (*P* < 0.05) (Table 2).

In the multivariate analysis, after adjusting for covariates, including gender, age, pathological classification, T-classification, N-classification, and M-classification, tumor location was no longer an independent factor for the prognosis of colorectal NENs (*P* = 0.08) (Table 2).

## 4. Discussion

The current study described the clinicopathology and assessed the prognostic factors among 68 cases of Chinese colorectal NENs. During the same period, 2460 colorectal adenocarcinoma patients registered to our center with complete follow-up information [17]. Colorectal NENs accounted for 2.8% of all the colorectal cancer patients. In clinical practice, colonic NENs usually present late, as large tumors, often with extensive metastatic disease and poor outcome. However, after multivariate analysis, we found that tumor size and pathological classification were independent prognostic factors, whereas tumor location was not.

Limited research is available regarding NENs, especially colorectal NENs. The rarity of NENs and lack of union definition are impediments to large-scale clinical trials and development of accepted guidelines for management. Although large population-based studies of gastrointestinal carcinoids have been conducted in the US, these studies did not focus on colorectal NENs or analyze the exact prognostic factors [5, 7, 9].

The inconsistent findings were noted among studies of NENs from different areas [5, 7, 9, 12, 14, 15]. Firstly, the proportion of rectal NENs varied widely. The current study revealed that 63.2% of colorectal NENs were located in the rectum. Our results were not consistent with previous report from Japan in which 304 of 345 (90%) cases of colorectal carcinoids originated from the rectum [12]. According to the SEER database [9], rectal carcinoids accounted for 31.6% of all the NENs in large intestine. In previous Chinese studies, Wang et al. [15] and Zhang et al. [14] collected clinical and pathological data from 178 and 168 patients diagnosed with gastroenteropancreatic neuroendocrine neoplasms (GEP-NENs), respectively, for analysis; these studies involve the largest NENs samples in China to date. Wang et al. [15] reported that the most common primary site was the pancreas (62/178, 34.8%) followed by rectum (36/178, 20.2%), stomach (25/178, 14.0%), duodenum (13/178, 7.3%), and unknown primary site (12/178, 6.7%). The ratio of colorectal NENs was only 20.8% (37/178). However, Zhang et al. [14] found that the rectum was the most common site of involvement (58.93%) followed by pancreas (13.69%), stomach (9.52%), duodenum (2.38%), colon (4.76%), and appendix (4.76%). In his study, colorectal NEN accounted for 63.69% of all patients. It is possible that some colonic carcinoids registered in SEER data were misdiagnosed and were actually poorly differentiated adenocarcinomas or undifferentiated carcinomas. Another possibility is the existence of an obviously increased frequency of both NENs and adenocarcinomas in right-sided colons with the westernized lifestyle [11, 18, 19]. In addition, the inconsistencies may be due to racial and geographical disparities. Overall, rectal NENs appear to be more common in the Asian population. In contrast, the prevalence of colonic and appendiceal NENs appears to be considerably increased among the Caucasian population.

In addition, a distinction in the aspect of distant metastasis was noted between colonic and rectal NENs. In the SEER database, 45% of colonic NENs were localized when diagnosed [9]. In a Japanese series, this ratio was 65% [12]. In current study, 48% of colonic NENs were localized when diagnosed. On the other hand, the majority of rectal NENs were localized at diagnosis. The ratios of metastatic rectal NEN patients were 5% in the SEER database [9], 8% in the Japanese registry [12], and 11.6% in the current study. Given that distant metastasis was one of the strongest prognostic factors of outcome [12, 20–24], differences in overall survival were also noted between colonic NENs and rectal NENs. In the SEER database, colonic NEN patients exhibited the worst prognosis among all GEP-NENs patients, with a 5-year overall survival rate of 41.6%. Rectal NENs appear to exhibit a low propensity to metastasize and thus are associated with a favorable prognosis, with a 5-year overall survival rate of 88.3% [9]. In current study, the 5-year survival rate for colonic NENs was 66.7%, and 5-year survival for rectal NENs was 88.1%, which was similar to that reported in the SEER database. In Wang et al.'s study, the 1-, 3-, and 5-year survival rates for GEP-NENs were 74.4%, 66.7%, and 54.5% [15], respectively, lower than the current study with a 5-year overall survival rate of 82.4% for colorectal NENs. This disparity was because the current cohort was exclusively composed of NENs originating from the colon and rectum, whereas the previous two studies contained GEP-NENs originating from any part of the digestive system.

Regarding the most important prognostic factors, each study did not arrive at exactly the same answer. Konishi et al. [12] studied 345 colorectal NENs cases and revealed that tumor site in the colon was statistically correlated with distant metastasis. However, after multivariate analysis, the independent risk factors for distant metastasis only included tumor size ≥ 21 mm and venous invasion. Tumor location was not an independent prognostic factor, and this finding is similar to the current results. Wang et al. [15] observed that NET/G1 patients without distant metastasis exhibited enhanced survival compared with patients with other types of NENs, thus suggesting that pathological classification, tumor grade, and distant metastasis were prognostic factors. However, the age, gender, and primary tumor location had little impact on overall survival. According to the study of Zhang et al. [14], the univariate analysis revealed that gender and tumor size were related to survival but did not translate into independent risk factors for survival according to the Cox regression model. Age and pathological classification were the only independent prognostic factors for overall survival (*P* = 0.02 and *P* = 0.04, resp.). Tumor location was not associated with prognosis (*P* = 0.11). In the current study, gender was not associated with prognosis, but tumor size and pathological classification were independent prognostic factors. Tumor location was associated with prognosis in the univariate analysis but not in the multivariate analysis. Given that numerous previous studies confirmed that GEP-NENs comprise a heterogeneous group in relation to their primary locations [2, 5, 9, 25], NENs originating from different sites should not be categorized in the same class. In previous Chinese studies of NENs, colorectal NENs were consistently grouped with GEP-NENs and underrepresented. Our study exclusively assessed colonic and rectal NENs, thus increasing the reliability of our study.

Tumor location was statistically significant in univariate analysis but not in multivariate analysis. Both univariate and multivariate analyses indicated that tumor size and pathological classification were independent factors for the prognosis of colorectal NENs. Based on the above results, we hypothesize that the worse outcome of colonic NENs observed in clinical practice might be due not only to the biological differences, but also to larger tumor size in colonic NENs caused by the delayed diagnosis. In other words, the association between tumor location and overall survival was influenced by tumor size and pathological classification. The diagnosis of colonic NENs at late stages was probably due to the lack of early performance and difficulty accessing high-quality endoscopy. Early detection enables tumor treatment at an earlier stage without distant metastasis, which is the key to achieving curative resection and prolonging survival. In summary, extending enteroscopy and the exploration of novel diagnostic methods were of the most importance to improve the prognosis of colonic NENs [26, 27].

Ki-67 is a kind of nucleus antigen reflecting cell proliferation, which closely associated with tumor proliferation, infiltration, metastasis potentiality, and prognosis. Previous researches have indicated that higher Ki-67 index showed worse prognosis in GEP-NENs [28, 29]. Some studies with small sample sizes of colorectal NENs also presented similar results. Based on the above main findings, ENETS Consensus Guidelines put forward that NENs could be graded as G1, G2, and G3 according to the Ki-67 index (G1, Ki-67 ≤ 2%; G2, Ki-67 3 to 20%; G3, Ki-67 > 20%) [8]. We could not evaluate the prognostic value of Ki-67 index due to incompleteness of data. In available data, the G1/G2 NET were in the majority, which was accordant to the related researches overseas.

Several limitations in this study should be noted. First, our data were retrospectively collected from single medical center, and this method carries an inherent risk of bias. Our data only included the Chinese population, and it is not clear whether the results could be generalized to populations worldwide. Second, additional known risk factors that could predict survival were not evaluated, including histological growth pattern, mitotic rate, Ki-67 index, and immunohistochemistry markers, such as chromogranin A (CgA) and synaptophysin (Syn) [21, 30–33]. Finally, our analysis did not adjust for treatment approaches that may impact outcomes [34]. To elucidate these questions, multicenter prospective studies with large samples are needed. Despite these limitations, we believe that current study reflects the actual distribution, clinical features, and prognostic factors of colorectal NENs in the Chinese population.

## 5. Conclusion

Significant differences in clinicopathological feature and outcome exist between colonic and rectal NENs. Multivariate analysis indicated that tumor size and pathological classification were associated with the prognosis. However, tumor location was not an independent factor. The worse outcome of colonic NENs observed in clinical practice might be due not only to the biological differences, but also to larger tumor size in colonic NENs caused by the delayed diagnosis.

## Figures and Tables

**Table 1:** The clinicopathological characteristics of 68 colorectal neuroendocrine neoplasm patients.

Variance	Rectal NEN (*n* = 43) (patients [%])	Colonic NEN (*n* = 25) (patients [%])	*P*
Gender			NS
Male	26 (60.5)	18 (72.0)	
Female	17 (39.5)	7 (28.0)	
Age			0.012
≤60	31 (72.1)	10 (40.0)	
>60	12 (27.9)	15 (60.0)	
Size (mm)			0.000
1–10	30 (69.8)	0 (0)	
11–20	6 (13.9)	2 (8.0)	
≥21	7 (16.3)	23 (92.0)	
T-classification^*∗*^			0.000
T1	26 (60.5)	0 (0)	
T2	9 (20.9)	1 (4.0)	
T3	4 (9.3)	12 (48.0)	
T4	4 (9.3)	12 (48.0)	
N-classification^*∗*^			0.000
N0	37 (86.0)	10 (40.0)	
N1	6 (14.0)	15 (60.0)	
M-classification^*∗*^			0.000
M0	38 (88.4)	12 (48.0)	
M1	5 (11.6)	13 (52.0)	
Stage			0.000
I	25 (58.1)	0 (0)	
II	9 (21.0)	4 (16.0)	
III	4 (9.3)	8 (32.0)	
IV	5 (11.6)	13 (52.0)	
Pathology^#^			0.001
NET	33 (76.7)	9 (36.0)	
NEC	8 (18.6)	8 (32.0)	
MANEC	2 (4.7)	8 (32.0)	
Ki-67 index (%)			NS
≤2	20 (46.5)	7 (28.0)	
3–20	4 (9.3)	2 (8.0)	
>20	8 (18.6)	8 (32.0)	
Unclear	11 (25.6)	8 (32.0)	

^*∗*^T-classification, N-classification, M-classification, and stages I, II, III, and IV according to the 7th AJCC TNM staging system.

^#^Pathological classification according to WHO 2010.

**Table 2:** Univariate and multivariate analysis (Cox proportional hazard model) of prognostic factors for 63 colorectal neuroendocrine neoplasm patients with complete follow-ups.

Variables (reference)	Univariate analysis			Multivariate analysis		
	RR	95% CI	*P*	RR	95% CI	*P*
Age (≤60 years old)	1.116	0.363–3.426	0.848	0.136	0.024–0.776	0.025
Gender (male)	0.947	0.285–3.147	0.929	1.533	0.389–6.045	0.541
Location (colon)	0.303	0.099–0.931	0.037	4.121	0.838–20.267	0.081
Size (≤10 mm)	3.801	1.491–9.692	0.005	0.088	0.009–0.859	0.037
Pathology (NET)	1.930	1.010–3.689	0.047	4.338	1.376–13.681	0.012
T-classification (T1)	4.730	1.946–11.502	0.001	25.326	3.919–163.668	0.001
N-classification (N0)	4.217	1.368–13.000	0.012	4.331	0.796–23.565	0.090
M-classification (M0)	24.761	5.357–114.441	0.000	33.791	4.486–254.524	0.001

RR: relative risk. CI: confident index.

## References

[1] Fraenkel M., Kim M., Faggiano A., de Herder W. W., Valk G. D. (2014). Incidence of gastroenteropancreatic neuroendocrine tumours: a systematic review of the literature. *Endocrine-Related Cancer*.

[2] Scherübl H., Streller B., Stabenow R. (2013). Clinically detected gastroenteropancreatic neuroendocrine tumors are on the rise: epidemiological changes in Germany. *World Journal of Gastroenterology*.

[3] Fraenkel M., Kim M. K., Faggiano A., Valk G. D. (2012). Epidemiology of gastroenteropancreatic neuroendocrine tumours. *Best Practice & Research: Clinical Gastroenterology*.

[4] Hallet J., Law C. H. L., Cukier M., Saskin R., Liu N., Singh S. (2015). Exploring the rising incidence of neuroendocrine tumors: a population-based analysis of epidemiology, metastatic presentation, and outcomes. *Cancer*.

[5] Yao J. C., Hassan M., Phan A. (2008). One hundred years after ‘carcinoid’: epidemiology of and prognostic factors for neuroendocrine tumors in 35,825 cases in the United States. *Journal of Clinical Oncology*.

[6] Lawrence B., Gustafsson B. I., Chan A., Svejda B., Kidd M., Modlin I. M. (2011). The epidemiology of gastroenteropancreatic neuroendocrine tumors. *Endocrinology and Metabolism Clinics of North America*.

[7] Maggard M. A., O'Connell J. B., Ko C. Y. (2004). Updated population-based review of carcinoid tumors. *Annals of Surgery*.

[8] Caplin M., Sundin A., Nillson O. (2012). ENETS consensus guidelines for the management of patients with digestive neuroendocrine neoplasms: colorectal neuroendocrine neoplasms. *Neuroendocrinology*.

[9] Modlin I. M., Lye K. D., Kidd M. (2003). A 5-decade analysis of 13,715 carcinoid tumors. *Cancer*.

[10] Modlin I. M., Latich I., Zikusoka M., Kidd M., Eick G., Chan A. K. C. (2006). Gastrointestinal carcinoids: the evolution of diagnostic strategies. *Journal of Clinical Gastroenterology*.

[11] Lin O. S., Kozarek R. A., Cha J. M. (2014). Impact of sigmoidoscopy and colonoscopy on colorectal cancer incidence and mortality: an evidence-based review of published prospective and retrospective studies. *Intestinal Research*.

[12] Konishi T., Watanabe T., Kishimoto J., Kotake K., Muto T., Nagawa H. (2007). Prognosis and risk factors of metastasis in colorectal carcinoids: results of a nationwide registry over 15 years. *Gut*.

[13] Ito T., Sasano H., Tanaka M. (2010). Epidemiological study of gastroenteropancreatic neuroendocrine tumors in Japan. *Journal of Gastroenterology*.

[14] Zhang X., Ma L., Bao H., Zhang J., Wang Z., Gong P. (2014). Clinical, pathological and prognostic characteristics of gastroenteropancreatic neuroendocrine neoplasms in China: a retrospective study. *BMC Endocrine Disorders*.

[15] Wang Y.-H., Lin Y., Xue L., Wang J.-H., Chen M.-H., Chen J. (2012). Relationship between clinical characteristics and survival of gastroenteropancreatic neuroendocrine neoplasms: a single-institution analysis (1995–2012) in South China. *BMC Endocrine Disorders*.

[16] Reed N. (2016). *ENETS conference for the diagnosis and treatment of neuroendocrine tumor disease*.

[17] Fu J., Yang J., Tan Y. (2014). Young patients (≤ 35 years old) with colorectal cancer have worse outcomes due to more advanced disease: a 30-year retrospective review. *Medicine*.

[18] Stewart S. L., Wike J. M., Kato I., Lewis D. R., Michaud F. (2006). A population-based study of colorectal cancer histology in the United States, 1998–2001. *Cancer*.

[19] Shen H., Yang J., Huang Q. (2015). Different treatment strategies and molecular features between right-sided and left-sided colon cancers. *World Journal of Gastroenterology*.

[20] Jiao X., Li Y., Wang H., Liu S., Zhang D., Zhou Y. (2015). Clinicopathological features and survival analysis of gastroenteropancreatic neuroendocrine neoplasms: a retrospective study in a single center of China. *Chinese Journal of Cancer Research*.

[21] de Miguel Novoa M. P., Fernández Capel F., Redondo Sedano J. V. (2014). Gastroenteropancreatic neuroendocrine tumors: clinical characteristics, diagnosis and prognosis at Hospital Universitario Clínico San Carlos (Madrid). *Endocrinologia y Nutricion*.

[22] Hu H.-K., Ke N.-W., Li A., Du X.-J., Guo Q., Hu W.-M. (2015). Clinical characteristics and prognostic factors of gastroenteropancreatic neuroendocrine tumors: a single center experience in China. *Hepato-Gastroenterology*.

[23] Lewkowicz E., Trofimiuk-Müldner M., Wysocka K. (2015). Gastroenteropancreatic neuroendocrine neoplasms: a 10-year experience of a single center. *Polskie Archiwum Medycyny Wewnetrznej*.

[24] Wang X., Song Z.-F., Yao W.-X., Pan C.-C., Xiang M.-F., Wang H. (2013). Clinicopathological features and multivariate analysis of prognostic factors for patients with gastroenteropancreatic neuroendocrine tumors. *Zhonghua Yi Xue Za Zhi*.

[25] Yucel B., Babacan N. A. K., Kacan T. (2014). Survival analysis and prognostic factors for neuroendocrine tumors in Turkey. *Asian Pacific journal of cancer prevention : APJCP*.

[26] Modlin I. M., Drozdov I., Alaimo D. (2014). A multianalyte PCR blood test outperforms single analyte ELISAs (chromogranin A, pancreastatin, neurokinin A) for neuroendocrine tumor detection. *Endocrine-Related Cancer*.

[27] Vicentini C., Fassan M., D'Angelo E. (2014). Clinical application of microRNA testing in neuroendocrine tumors of the gastrointestinal tract. *Molecules*.

[28] Boo Y.-J., Park S.-S., Kim J.-H., Mok Y.-J., Kim S.-J., Kim C.-S. (2007). Gastric neuroendocrine carcinoma: clinicopathologic review and immunohistochemical study of E-cadherin and Ki-67 as prognostic markers. *Journal of Surgical Oncology*.

[29] Miller H. C., Drymousis P., Flora R., Goldin R., Spalding D., Frilling A. (2014). Role of ki-67 proliferation index in the assessment of patients with neuroendocrine neoplasias regarding the stage of disease. *World Journal of Surgery*.

[30] Massironi S., Rossi R. E., Casazza G. (2014). Chromogranin a in diagnosing and monitoring patients with gastroenteropancreatic neuroendocrine neoplasms: a large series from a single institution. *Neuroendocrinology*.

[31] Stridsberg M., Oberg K., Li Q., Engstrom U., Lundqvist G. (1995). Measurements of chromogranin A, chromogranin B (secretogranin I), chromogranin C (secretogranin II) and pancreastatin in plasma and urine from patients with carcinoid tumours and endocrine pancreatic tumours. *The Journal of Endocrinology*.

[32] Jernman J., Hagström J., Mäenpää H. (2015). Expression of stem cell-associated marker HES77 in rectal neuroendocrine tumors. *Anticancer Research*.

[33] Wang Y.-H., Yang Q.-C., Lin Y., Xue L., Chen M.-H., Chen J. (2014). Chromogranin a as a marker for diagnosis, treatment, and survival in patients with gastroenteropancreatic neuroendocrine neoplasm. *Medicine*.

[34] Pusceddu S., De Braud F., Festinese F. (2015). Evolution in the treatment of gastroenteropancreatic-neuroendocrine neoplasms, focus on systemic therapeutic options: a systematic review. *Future Oncology*.

